# Analysis of patients without and with an initial triple-negative breast cancer diagnosis in the phase 3 randomized ASCENT study of sacituzumab govitecan in metastatic triple-negative breast cancer

**DOI:** 10.1007/s10549-022-06602-7

**Published:** 2022-05-11

**Authors:** Joyce O’Shaughnessy, Adam Brufsky, Hope S. Rugo, Sara M. Tolaney, Kevin Punie, Sagar Sardesai, Erika Hamilton, Delphine Loirat, Tiffany Traina, Roberto Leon-Ferre, Sara A. Hurvitz, Kevin Kalinsky, Aditya Bardia, Stephanie Henry, Ingrid Mayer, Yanni Zhu, See Phan, Javier Cortés

**Affiliations:** 1 Medical Oncology, Texas Oncology-Baylor Charles A. Sammons Cancer Center, 3410 Worth St., Suite 400, Dallas, TX 75246 USA; 2 Magee-Womens Hospital and the Hillman Cancer Center, University of Pittsburgh Medical Center, Pittsburgh, PA USA; 3 Department of Medicine, University of California San Francisco Helen Diller Family Comprehensive Cancer Center, San Francisco, CA USA; 4 Medical Oncology, Dana-Farber Cancer Institute, Boston, MA USA; 5 Department of General Medical Oncology and Multidisciplinary Breast Centre, Leuven Cancer Institute, University Hospitals Leuven, Leuven, Belgium; 6 The Ohio State University Comprehensive Cancer Center, Columbus, OH USA; 7 Sarah Cannon Research Institute/Tennessee Oncology, Nashville, TN USA; 8 Medical Oncology Department and D3i, Institut Curie, Paris, France; 9 Memorial Sloan Kettering Cancer Center, New York, NY USA; 10 Department of Oncology, Mayo Clinic, Rochester, MN USA; 11 Medical Oncology, University of California, Los Angeles, Jonsson Comprehensive Cancer Center, Los Angeles, CA USA; 12 Columbia University Irving Medical Center, New York, NY USA; 13 Department of Hematology/Oncology, Massachusetts General Hospital Cancer Center, Harvard Medical School, Boston, USA; 14 Department of Oncology-Hematology, Radiotherapy, and Nuclear Medicine, CHU UCL Namur, Namur, Belgium; 15 Breast Cancer Program, Division of Hematology/Oncology, Vanderbilt-Ingram Cancer Center, Nashville, TN USA; 16 Department of Biostatistics, Gilead Sciences, Inc., Foster City, CA USA; 17 Department of Clinical Development, Gilead Sciences, Inc., Foster City, CA USA; 18 International Breast Cancer Center, Quironsalud Group, Barcelona, Spain; 19 Present Address: Winship Cancer Institute, Emory University, Atlanta, GA USA

**Keywords:** Sacituzumab govitecan, Antibody–drug conjugate, Cyclin-dependent kinase inhibitor

## Abstract

**Purpose:**

Sacituzumab govitecan (SG) is an antibody–drug conjugate composed of an anti–Trop-2 antibody coupled to SN-38 via a proprietary hydrolyzable linker. In the ASCENT study, SG improved survival versus single-agent treatment of physician’s choice (TPC) in pre-treated metastatic triple-negative breast cancer (mTNBC). Hormone/HER2 receptor changes are common, particularly at relapse/metastasis. This subanalysis assessed outcomes in patients who did/did not have TNBC at initial diagnosis, before enrollment.

**Methods:**

TNBC diagnosis was only required at study entry. Patients with mTNBC refractory/relapsing after ≥ 2 prior chemotherapies were randomized 1:1 to receive SG or TPC. Primary endpoint was progression-free survival (PFS) in patients without brain metastases.

**Results:**

Overall, 70/235 (30%) and 76/233 (33%) patients who received SG and TPC, respectively, did not have TNBC at initial diagnosis. Clinical benefit with SG versus TPC was observed in this subset. Median PFS was 4.6 versus 2.3 months (HR 0.48; 95% CI 0.32–0.72), median overall survival was 12.4 versus 6.7 months (HR 0.44; 95% CI 0.30–0.64), and objective response rate (ORR) was 31% versus 4%; those who also received prior CDK4/6 inhibitors had ORRs of 21% versus 5%. Efficacy and safety for patients with TNBC at initial diagnosis were generally similar to those who did not present with TNBC at initial diagnosis.

**Conclusion:**

Patients without TNBC at initial diagnosis had improved clinical outcomes and a manageable safety profile with SG, supporting SG as a treatment option for mTNBC regardless of subtype at initial diagnosis. Subtype reassessment in advanced breast cancer allows for optimal treatment.

*Clinical trial registration number* NCT02574455, registered October 12, 2015.

**Supplementary Information:**

The online version contains supplementary material available at 10.1007/s10549-022-06602-7.

## Introduction

Approximately 15% of breast cancers diagnosed each year are categorized as triple-negative [1]. This subtype is defined by its combined lack of human epidermal growth factor receptor 2 (HER2) amplification, estrogen-receptor (ER) expression, and progesterone-receptor (PR) expression [2–4]. Challenges in treating TNBC include its aggressive behavior and heterogeneity, and limited viable targets and effective targeted therapies [2–5]. Standard of care for pre-treated metastatic TNBC (mTNBC) remains single-agent chemotherapy, such as eribulin, and most patients receive multiple lines of therapy in the metastatic setting [6]. However, progression-free survival (PFS) and response rates to later-line therapies are low and associated with significant toxicity, underscoring the need for novel therapies [7–10].

A barrier toward optimizing clinical outcomes for breast cancer is receptor status discordance. Although the majority (88%) of patients with breast cancer are initially diagnosed with hormone receptor (Hr)-positive and/or human epidermal growth factor receptor 2 (HER2)-positive disease [11, 12], receptor status discordance from breast cancer diagnosis through relapses and disease progression occurs frequently, most commonly involving changes in Hr status [13–17]. One retrospective analysis of 993 intraindividual tissue samples from primary breast tumors and relapses found alterations in estrogen receptor (ER), progesterone receptor (PR), and HER2 status in 32%, 41%, and 15% of patients, respectively [15]. Positive-to-negative changes in receptor status occur more frequently than negative-to-positive changes [13, 15], with implications for clinical outcomes. Loss of ER, PR, or HER2 expression between primary and recurrent tumors is associated with poorer overall survival (OS) and post-recurrence survival compared with receptor stability between primary and recurrent tumors [14–16]. Recognizing the impact on prognosis, tissue confirmation of recurrent/metastatic breast cancer subtype is included in breast cancer management guidelines [6, 18]. However, definitive, evidence-based guidance on treatment decision-making in the setting of discordant receptor status is lacking.

Sacituzumab govitecan is a Trop-2–directed antibody–drug conjugate composed of a humanized anti-Trop-2 IgG_1_ kappa antibody coupled to SN-38, the active metabolite of the topoisomerase inhibitor irinotecan, via a proprietary, hydrolyzable linker [19–21]. Following SG administration, the anti–Trop-2 monoclonal antibody binds to Trop-2 expressed on the tumor cell surface, enabling SN-38 internalization and targeted delivery to tumor cells [19, 22]. Its proprietary linker allows SN-38 to be liberated in the tumor microenvironment, eliciting antitumor effects (bystander effect) without prerequisite internalization and enzymatic cleavage of SN-38 from the anti-Trop-2 antibody [19, 22, 23].

In a phase 1/2, single-arm, basket study (IMMU-132-01; NCT01631552), SG was evaluated for patients with metastatic, epithelial cancers. In this study, a cohort of 108 patients with heavily pre-treated mTNBC treated with SG reported an ORR of 33%, a clinical benefit rate (CBR) of 45%, a median PFS of 5.5 months, a median OS of 13.0 months, and a manageable safety profile [24]. These results led to accelerated approval of SG by the United States Food and Drug Administration (FDA), with full approval received based on results of the randomized phase 3 ASCENT study [25].

The phase 3 ASCENT study evaluated the efficacy and safety of SG compared with single-agent treatment of physician’s choice (TPC; eribulin, vinorelbine, gemcitabine, or capecitabine) in 529 patients with pre-treated mTNBC. Results from this trial confirmed the initial findings from the phase 1/2 study. In the primary efficacy population of 468 patients without known brain metastasis, SG significantly improved survival compared with TPC, with a median PFS of 5.6 months versus 1.7 months (hazard ratio [HR] 0.41; [95% confidence interval [CI] 0.3–0.5]; *p* < 0.001) and a median OS of 12.1 months versus 6.7 months (HR 0.48; 95% CI 0.38–0.59; *p* < 0.001) [26]. PFS and OS benefit for SG was consistently observed across all predefined subgroups, and SG demonstrated a manageable safety profile [26].

Patients in ASCENT were required to have TNBC only at study entry; therefore, ASCENT included patients who may have had an initial diagnosis of another breast cancer subtype, such as Hr/HER2-positive disease. Because the ASCENT study population is heavily pre-treated, altered receptor status over the disease course may have been common among these patients. In this exploratory subgroup analysis of data from ASCENT, we assess the clinical impact of SG in the subgroup of patients who did not have TNBC at initial diagnosis.

## Patients and methods

### Study design

Full details of the study design for ASCENT (NCT02574455) have been described previously [26]. Briefly, patients with pre-treated mTNBC were randomized 1:1 to receive SG (10 mg/kg on days 1 and 8 of 21-day cycles) or TPC (eribulin, vinorelbine, gemcitabine, or capecitabine) until progression, unacceptable toxicity, study withdrawal, or death. The primary endpoint was PFS by blinded independent central review (BICR) in patients without known baseline brain metastases (BMNeg) per Response Evaluation Criteria in Solid Tumors (RECIST) version 1.1. Secondary endpoints included investigator-assessed PFS, OS, ORR (per RECIST 1.1), duration of response (DOR), and safety.

The ASCENT trial was conducted and approved by each investigational site’s institutional review board/ethics committee prior to initiation, and in accordance with the Declaration of Helsinki, International Council for Harmonisation Good Clinical Practice Guidelines, FDA Code of Federal Regulations, national and local drug and data protection laws, and other applicable regulatory requirements. All patients provided written informed consent before enrollment.

### Patients

Patients had mTNBC that had progressed following ≥ 2 prior standard chemotherapy regimens (no upper limit) for unresectable, locally advanced, or metastatic disease, and included a taxane (any setting). Per protocol, patients were also eligible after only one prior regimen in the metastatic setting if their disease recurred within 12 months of completing (neo)adjuvant therapy. TNBC status at initial diagnosis was determined from patient histories; biopsies at initial diagnosis were not centrally assessed for this study. TNBC/receptor status prior to enrollment in ASCENT was determined by local assessment of most recent biopsy or other pathology specimen per American Society of Clinical Oncology/College of American Pathologists criteria [27, 28]. Negativity for ER and PR was defined as < 1% of cells expressing ER or PR by immunohistochemistry (IHC). Negativity for HER2 was defined as IHC0 or IHC1+, or if IHC2+, then fluorescence in situ hybridization negative. Collection of new tissue samples after disease metastasis was not required for ASCENT.

### Statistical analysis

This post hoc subanalysis evaluated efficacy and safety outcomes for the subpopulations of patients with and without TNBC at their initial breast cancer diagnosis. Efficacy outcomes in these subgroups were assessed in BMNeg patients. Median PFS and ORR were assessed by BICR per RECIST 1.1. The Kaplan–Meier method was used to analyze median PFS and OS. Hazard ratios and 95% confidence intervals were estimated using an unstratified Cox regression model. Safety outcomes were assessed in all patients (with and without brain metastases) who received one or more doses of study treatment. Adverse events (AEs) were coded using the Medical Dictionary for Regulatory Activities v22.1, and AE severity was graded per National Cancer Institute Common Terminology Criteria v4.03. Data cutoff for this analysis was March 11, 2020.

## Results

### Patients

Between November 2017 and September 2019, 529 patients with TNBC were enrolled in ASCENT; 468 had no evidence of brain metastases at baseline. A total of 146 BMNeg patients did not have TNBC at initial diagnosis (70/235 [30%] patients in the SG arm and 76/233 [33%] in the TPC arm). The disposition of patients without and with TNBC at initial diagnosis in ASCENT is summarized in Online Resource 1. Demographic and baseline characteristics of patients without and with TNBC at initial diagnosis (Table 1) were generally balanced across treatment arms and comparable in patients without and with TNBC at initial diagnosis. The median age in patients without TNBC at initial diagnosis was 56 years (range 31–74) and 55 years (range 27–80) for patients in the SG and TPC arms, respectively. Patients without TNBC at initial diagnosis received a median of 5 prior anticancer regimens (defined as any treatment regimen used to treat breast cancer in any setting, including endocrine therapy and any targeted treatment), whereas patients with TNBC at initial diagnosis received a median of four prior anticancer regimens.

**Table 1:** Demographics and baseline characteristics of patients without and with TNBC at initial diagnosis

	Patients without TNBC at initial diagnosis		Patients with TNBC at initial diagnosis	
	SG (*n* = 70)	TPC (*n* = 76)	SG (*n* = 165)	TPC (*n* = 157)
Female, *n* (%)	69 (99)	76 (100)	164 (99)	157 (100)
Median age, years (range)	56 (31–74)	55 (27–80)	54 (29–82)	52 (31–81)
Race or ethnic group, *n* (%)				
White	58 (83)	62 (82)	130 (79)	119 (76)
Black	6 (9)	5 (7)	22 (13)	23 (15)
Asian	3 (4)	4 (5)	6 (4)	5 (3)
Other or not specified	3 (4)	5 (7)	7 (4)	10 (6)
ECOG PS, *n* (%)				
0	28 (40)	26 (34)	80 (48)	72 (46)
1	42 (60)	50 (66)	85 (52)	85 (54)
Number of prior chemotherapies for stratification, *n* (%)				
2–3	41 (59)	46 (61)	125 (76)	118 (75)
> 3	29 (41)	30 (39)	40 (24)	39 (25)
Median prior anticancer regimens,^a^ *n* (range)	5 (2–17)	5 (2–14)	4 (2–11)	4 (2–10)
Previous use of checkpoint inhibitor, *n* (%)	17 (24)	23 (30)	50 (30)	37 (24)
Previous use of CDK4/6 inhibitor, *n* (%)	19 (27)	22 (29)	3 (2)	2 (1)
Previous use of anti-HER2 therapy, *n* (%)	14 (20)	13 (17)	7 (4)	7 (4)
Previous use of PI3K inhibitors,^b^ *n* (%)	2 (3)	0	2 (1)	2 (1)
Previous use of PARP inhibitors, *n* (%)	4 (6)	5 (7)	13 (8)	13 (8)
Setting of prior systemic therapies, *n* (%)				
Adjuvant	54 (77)	55 (72)	86 (52)	74 (47)
Neoadjuvant	30 (43)	30 (39)	83 (50)	81 (52)
Metastatic	69 (99)	76 (100)	157 (95)	155 (99)
Locally advanced disease	2 (3)	1 (1)	6 (4)	3 (2)
ER < 1% of tumor cells, *n* (%)	70 (100)	76 (100)	165 (100)	157 (100)
PR < 1% of tumor cells, *n* (%)	70 (100)	76 (100)	165 (100)	157 (100)
Diagnosis of HER2 negativity, *n* (%)				
IHC 0	31 (44)	37 (49)	99 (60)	88 (56)
IHC 1	16 (23)	13 (17)	25 (15)	29 (18)
FISH	23 (33)	26 (34)	41 (25)	40 (25)
*BRCA1/2* mutational status, *n* (%)				
Negative	43 (61)	36 (47)	90 (55)	89 (57)
Positive	6 (9)	4 (5)	10 (6)	14 (9)
Trop-2 expression, *n* (%)				
(High) *H*-score > 200–300	27 (39)	22 (29)	58 (35)	50 (32)
(Medium) *H*-score 100–200	12 (17)	13 (17)	27 (16)	22 (14)
(Low) *H*-score 0 to < 100	7 (10)	7 (9)	20 (12)	25 (16)

Assessed in the brain metastasis-negative population

*BRCA* breast cancer gene; *CDK* cyclin-dependent kinase; *ECOG PS* Eastern Cooperative Oncology Group performance status; *ER* estrogen receptor; *FISH* fluorescence in situ hybridization; *HER2* human epidermal growth factor receptor 2; *H-score* histological score; *IHC* immunohistochemistry; *PARP* poly (adenosine diphosphate-ribose) polymerase; *PI3K* phosphoinositide 3 kinase; *PR* progesterone receptor; *SG* sacituzumab govitecan; *TNBC* triple-negative breast cancer; *TPC* treatment of physician’s choice

^a^Anticancer regimens refer to any treatment regimen that was used to treat breast cancer in any setting and includes endocrine therapy and everolimus

^b^Previous everolimus use is not counted under previous PI3K inhibitor use

In patients without TNBC at initial diagnosis, 24% and 27% received prior immune checkpoint inhibitor and cyclin-dependent kinase (CDK) 4/6 inhibitor therapy in the SG arm, respectively; in the TPC arm, 30% and 29% of patients received prior immune checkpoint inhibitor and CDK4/6 inhibitor therapy, respectively (Table 1). In the SG versus TPC arms, 20% versus 17%, 3% versus 0%, and 6% versus 7% of patients received prior anti-HER2, phosphoinositide 3 kinase (PI3K) inhibitor, and poly (ADP-ribose) polymerase (PARP) inhibitor therapy, respectively. Compared with patients without TNBC at initial diagnosis, those with TNBC at initial diagnosis had similar frequencies of prior immune checkpoint, PI3K, and PARP inhibitor use, but lower frequencies of prior CDK4/6 inhibitor (2% and 1%) and anti-HER2 therapy (4% and 4%) use in both the SG and TPC arms, respectively.

In patients without TNBC at initial diagnosis, 4 patients (6%) in the SG arm remained on treatment at data cutoff, whereas no patients remained on treatment in the TPC arm. Most patients in both the SG (84%) and TPC (72%) arms discontinued due to progressive disease. In the SG arm, 3 patients each (4% each) discontinued due to AEs and physician decision. In the TPC arm, 5 (7%), 2 (3%), and 1 (1%) patient(s) discontinued due to withdrawal of consent, AEs, and death, respectively. Patients without TNBC at initial diagnosis had a median treatment duration of 5.1 months with SG and 1.2 months with TPC.

### Efficacy outcomes

**Fig. 1:**
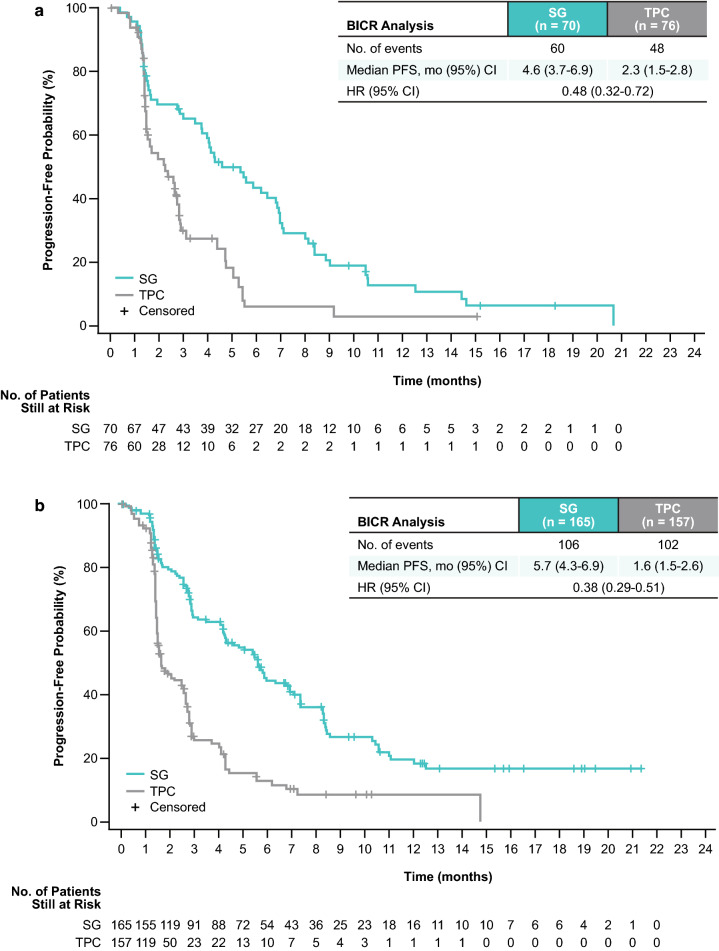
Kaplan–Meier estimates of progression-free survival are shown for patients without TNBC at initial diagnosis (**a**) and with TNBC at initial diagnosis (**b**). Assessments were in the brain metastases-negative population. *BICR* blinded independent central review; *CI* confidence interval; *HR* hazard ratio; *PFS* progression-free survival; *SG* sacituzumab govitecan; *TNBC* triple-negative breast cancer; *TPC* treatment of physician’s choice

As previously reported, efficacy outcomes were consistently improved in the SG versus TPC arms for all predefined subgroups [26]. At a median follow-up of 8.2 months (range 0.0–23.0), the median PFS by BICR for patients without TNBC at initial diagnosis was 4.6 months for SG versus 2.3 months for TPC (HR 0.48; 95% CI 0.32–0.72; Fig. 1a). In this subgroup, the 12-month PFS rate for patients treated with SG versus TPC was 13% (95% CI 5.7–22.8) versus 3% (95% CI 0.2–13.2). In patients with TNBC at initial diagnosis, median PFS was 5.7 versus 1.6 months for SG versus TPC (HR 0.38; 95% CI 0.29–0.51; Fig. 1b); the 12-month PFS rate was 20% (95% CI 12.5–27.8) versus 9% (95% CI 3.8–15.9). Improvements in PFS for patients without TNBC at initial diagnosis were similar to those observed in the total population of randomized patients, who had a median PFS of 4.8 versus 1.7 months (HR 0.43; 95% CI 0.35–0.54) [26], and a 12-month PFS rate of 16% (95% CI 11.2–22.0) versus 6% (95% CI 2.7–11.2).

**Fig. 2:**
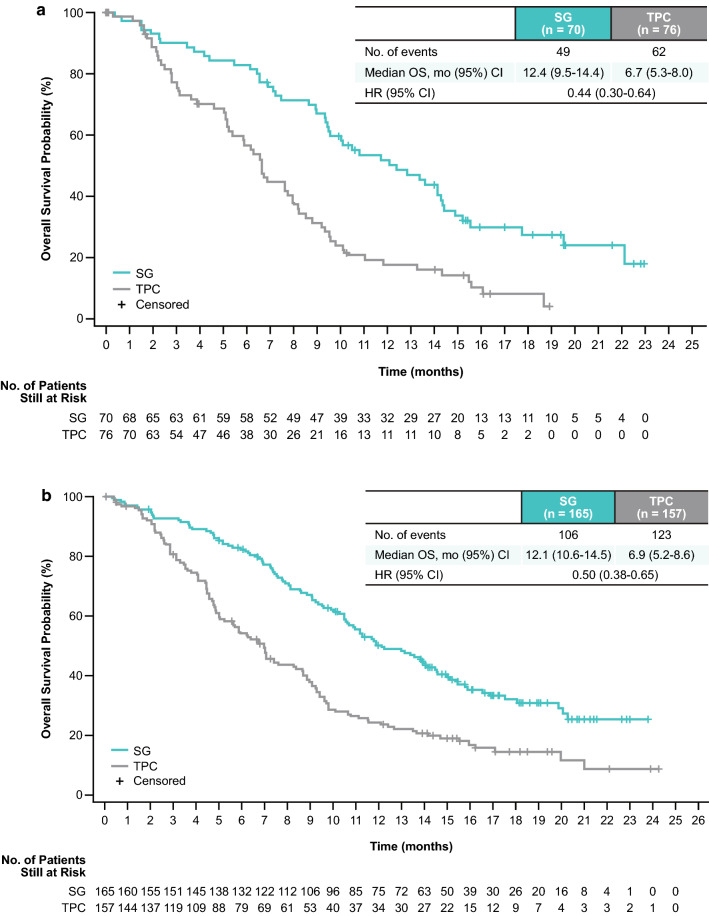
Kaplan–Meier estimates of overall survival are shown for patients without TNBC at initial diagnosis (**a**) and with TNBC at initial diagnosis (**b**). Assessments were in the brain metastases-negative population. *CI* confidence interval; *HR* hazard ratio; *OS* overall survival; *SG* sacituzumab govitecan; *TNBC* triple-negative breast cancer; *TPC* treatment of physician’s choice

In patients without TNBC at initial diagnosis, the median OS was 12.4 months for SG versus 6.7 months for TPC (HR 0.44; 95% CI 0.30–0.64; Fig. 2a); the 12- and 18-month OS rates were 52% (95% CI 39.3–62.9) versus 18% (9.6–27.7) and 27% (95% CI 16.6–39.1) versus 8% (95% CI 2.9–17.1), respectively. In patients with TNBC at initial diagnosis, median OS was 12.1 versus 6.9 months for SG versus TPC (HR 0.50; 95% CI 0.38–0.65; Fig. 2b); the 12- and 18-month OS rates were 50% (95% CI 42.2–57.7) versus 24% (95% CI 17.6–31.6) and 32% (95% CI 24.4–40.1) versus 15% (95% CI 8.9–21.4), respectively. OS improvements for patients without TNBC at initial diagnosis with SG versus TPC were similar to those observed in the total population of randomized patients, who had a median OS of 11.8 versus 6.9 months (HR 0.51; 95% CI 0.41–0.62) [26]; 12- and 18-month OS rates were 49% (95% CI 42.5–54.8) versus 23% (95% CI 17.8–28.5) and 29% (95% CI 22.6–34.8) versus 13% (95% CI 8.7–18.0) in the total population of randomized patients.

In patients without TNBC at initial diagnosis, the ORR was 31% in the SG arm versus 4% in the TPC arm. In the SG arm, 1 patient (1%) had a complete response (CR); 21 patients (30%) had a partial response (PR; Table 2). In the TPC arm, 1 patient (1%) had a CR, and 2 patients (3%) had a PR. In the SG versus TPC arms, the CBR was 44% versus 7%, and median DOR was 5.6 versus 3.5 months, respectively. Response outcomes were similar in patients with TNBC at initial diagnosis; in the SG versus TPC arms, 9 patients (5%) versus 1 patient (1%) had a CR, and 51 patients (31%) versus 7 patients (4%) had a PR.

**Table 2:** Clinical efficacy in patients without and with TNBC at initial diagnosis

	Patients without TNBC at initial diagnosis		Patients with TNBC at initial diagnosis	
	SG (*n* = 70)	TPC (*n* = 76)	SG (*n* = 165)	TPC (*n* = 157)
Median PFS, mo (95%) CI	4.6 (3.7–6.9)	2.3 (1.5–2.8)	5.7 (4.3–6.9)	1.6 (1.5–2.6)
HR (95% CI)	0.48 (0.32–0.72)		0.38 (0.29–0.51)	
Median OS, mo (95%) CI	12.4 (9.5–14.4)	6.7 (5.3–8.0)	12.1 (10.6–14.5)	6.9 (5.2–8.6)
HR (95% CI)	0.44 (0.30–0.64)		0.50 (0.38–0.65)	
ORR, *n* (%)	22 (31)	3 (4)	60 (36)	8 (5)
Best overall response, *n* (%)				
CR	1 (1)	1 (1)	9 (5)	1 (1)
PR	21 (30)	2 (3)	51 (31)	7 (4)
SD	26 (37)	24 (32)	55 (33)	38 (24)
SD > 6 months	9 (13)	2 (3)	14 (8)	7 (4)
PD	18 (26)	24 (32)	36 (22)	65 (41)
Not evaluable	4 (6)	25 (33)	14 (8)	46 (29)
CBR,^a^ *n* (%)	31 (44)	5 (7)	74 (45)	15 (10)
Median DOR, mo (95% CI)	5.6 (4.2–9.0)	3.5 (2.9–4.2)	7.1 (5.5–9.3)	NE (2.8-NE)

Assessed by independent central review in the brain metastasis-negative population

*CBR* clinical benefit rate; *CR* complete response; *DOR* duration of response; *HR* hazard ratio; *mo* months; *NE* not evaluable; *ORR* objective response rate; *OS* overall survival; *PD* progressive disease; *PFS* progression-free survival; *PR* partial response; *SD* stable disease; *SG* sacituzumab govitecan; *TPC* treatment of physician’s choice

^a^CBR is defined as the percentage of patients with a confirmed best overall response of CR, PR, and SD ≥ 6 months

Among patients without TNBC at initial diagnosis and who had received a prior CDK4/6 inhibitor, patients who received SG (*n* = 19) had numerically higher response rates versus those who received TPC (*n* = 22; 21% vs. 5%; Table 3). In the SG and TPC arms, 4 patients (21%) and 1 patient (5%) had a PR as the best overall response, respectively. In the SG versus TPC arms, the CBR was 32% versus 5%.

**Table 3:** Treatment response in patients without TNBC at initial diagnosis who received prior CDK4/6 inhibitor

	SG (*n* = 19)	TPC (*n* = 22)
ORR, *n* (%)	4 (21)	1 (5)
Best overall response, *n* (%)		
CR	0	0
PR	4 (21)	1 (5)
SD	10 (53)	6 (27)
SD > 6 months	2 (11)	0
PD	3 (16)	7 (32)
Not evaluable	2 (11)	8 (36)
CBR,^a^ *n* (%)	6 (32)	1 (5)

Assessed by independent central review in the brain metastasis-negative population

*CBR* clinical benefit rate; *CR* complete response; *ORR* objective response rate; *PD* progressive disease; *PR* partial response; *SD* stable disease; *SG* sacituzumab govitecan; *TPC* treatment of physician’s choice

^a^CBR is defined as the percentage of patients with a confirmed best overall response of CR, PR, and SD ≥ 6 months

### Safety outcomes

In patients without TNBC at initial diagnosis, the most common treatment-related AEs (TRAE) of any grade for SG versus TPC were neutropenia (73% vs. 47%), diarrhea (62% vs. 12%), nausea (62% vs. 26%), alopecia (47% vs. 9%), fatigue (50% vs. 32%), and anemia (31% vs. 25%), respectively (Table 4). The most common grade ≥ 3 TRAEs in the SG versus TPC arms were neutropenia (59% vs. 40%), leukopenia (12% vs. 9%), anemia (8% vs. 7%), and diarrhea (7% vs. 0%) in patients without TNBC at initial diagnosis. Key TRAEs were generally similar for patients with TNBC at initial diagnosis. In patients without TNBC at initial diagnosis, 2 patients in each arm (each 3%) experienced grade ≥ 3 treatment-related febrile neutropenia; in those with TNBC at initial diagnosis, 13 (7%) and three (2%) patients had grade ≥ 3 treatment-related febrile neutropenia in the SG versus TPC arms, respectively. In patients without and with TNBC at initial diagnosis, treatment-related peripheral neuropathy of any grade was observed in 3 (4%) versus 9 (13%) patients and 6 (3%) versus 18 (12%) patients in the SG versus TPC arms, respectively; grade ≥ 3 peripheral neuropathy was observed in zero versus 2 (3%) patients in the group without TNBC at initial diagnosis and zero versus 2 (1%) patients in the group with TNBC at initial diagnosis, respectively. In patients without and with TNBC at initial diagnosis, 2 events (3%) versus no events and 4 events (2%) versus 1 event (1%) of grade ≤ 2 treatment-related electrocardiogram QT prolonged (by preferred term) occurred in the SG versus TPC arms, respectively; no grade ≥ 3 treatment-related events of electrocardiogram QT prolonged occurred in either treatment arm. In patients without TNBC at initial diagnosis, no events of treatment-related interstitial lung disease occurred in either arm; in those with TNBC at initial diagnosis, 1 pneumonitis event occurred (grade 3, 1%) in the SG arm that resolved after drug withdrawal.

**Table 4:** TRAEs any grade (≥ 20%) and grade ≥ 3 (≥ 5%) in patients without and with TNBC at initial diagnosis

TRAE,^a^ *n* (%)		Patients without TNBC at initial diagnosis						Patients with TNBC at initial diagnosis					
		SG (*n* = 74)			TPC (*n* = 68)			SG (*n* = 184)			TPC (*n* = 156)		
		All grade	Grade 3	Grade 4	All grade	Grade 3	Grade 4	All grade	Grade 3	Grade 4	All grade	Grade 3	Grade 4
Hematologic	Neutropenia^b^	54 (73)	28 (38)	16 (22)	32 (47)	17 (25)	10 (15)	109 (59)	60 (33)	28 (15)	64 (41)	28 (18)	19 (12)
	Anemia^c^	23 (31)	6 (8)	0	17 (25)	5 (7)	0	66 (36)	14 (8)	0	37 (24)	6 (4)	0
	Leukopenia^d^	12 (16)	8 (11)	1 (1)	10 (15)	4 (6)	2 (3)	29 (16)	15 (8)	2 (1)	15 (10)	6 (4)	0
	Febrile neutropenia	2 (3)	2 (3)	0	2 (3)	2 (3)	0	13 (7)	10 (5)	3 (2)	3 (2)	2 (1)	1 (1)
Gastrointestinal	Nausea	46 (62)	2 (3)	0	18 (26)	1 (1)	0	101 (55)	4 (2)	1 (1)	41 (26)	0	0
	Diarrhea	46 (62)	5 (7)	0	8 (12)	0	0	107 (58)	22 (12)	0	19 (12)	1 (1)	0
	Vomiting	22 (30)	0	0	7 (10)	1 (1)	0	53 (29)	2 (1)	1 (1)	16 (10)	0	0
Other	Fatigue	37 (50)	1 (1)	0	22 (32)	5 (7)	0	78 (42)	7 (4)	0	46 (29)	7 (4)	0
	Alopecia	35 (47)	0	0	6 (9)	0	0	84 (46)	0	0	29 (19)	0	0
	Decreased appetite	19 (26)	0	0	12 (18)	0	0	32 (17)	4 (2)	0	20 (13)	1 (1)	0

Assessed in the safety population

*AE* adverse event; *MedDRA* Medical Dictionary for Regulatory Activities; *NCI CTCAE* National Cancer Institute Common Terminology Criteria for AE; *SG* sacituzumab govitecan; *TPC* treatment of physician’s choice; *TRAE* treatment-related AE

^a^Patients may report more than one event per preferred term. AEs were coded using MedDRA v22.1, and AE severity was graded per NCI CTCAE v4.03

^b^Combined preferred terms of ‘neutropenia’ and ‘neutrophil count decreased’

^c^Combined preferred terms of ‘anemia,’ ‘hemoglobin decreased,’ and ‘red blood cell count decreased’

^d^Combined preferred terms of ‘leukopenia’ and ‘white blood cell count decreased’

In patients without TNBC at initial diagnosis, 16% and 25% of patients in the SG and TPC arms, respectively, had dose reductions due to TRAEs; the most common reasons for dose reduction were neutropenia (9% and 25%) and diarrhea (4% and 0%). Discontinuations due to treatment-emergent AEs were low for SG and TPC (5% and 7%, respectively), and no treatment-related deaths occurred in either arm in this subgroup. In patients with TNBC at initial diagnosis, the frequency of dose reductions due to TRAEs in the SG versus TPC arms was similar (21% vs. 22%); the most common reason for dose reduction was neutropenia (11% vs. 17%, including both neutropenia and febrile neutropenia). Discontinuations due to treatment-emergent AEs were low for both arms (4% for both) in this subgroup. One treatment-related death occurred in the TPC arm for this subgroup.

## Discussion

The pivotal phase 3 randomized ASCENT trial demonstrated improvement in PFS, OS, and ORR with SG compared with TPC (eribulin, vinorelbine, gemcitabine, or capecitabine) in patients with heavily pre-treated metastatic TNBC [26]. Due to the eligibility criteria, the overall study population of ASCENT included patients without TNBC at initial diagnosis. In the current subanalysis of ASCENT, the clinical benefit of SG over TPC was confirmed in patients who did not have TNBC at initial breast cancer diagnosis; this benefit was similar to that observed for the ASCENT primary analysis population of all randomized BMNeg patients and the total ASCENT study population [26]. Key efficacy outcomes with the use of SG versus TPC for this subgroup were a median PFS of 4.6 versus 2.3 months, median OS of 12.4 versus 6.7 months, and ORR of 31% versus 4%. Responses were durable with SG versus TPC, with a median DOR of 5.6 versus 3.5 months. SG also had a manageable safety profile in patients without TNBC at initial diagnosis, which was generally similar to that of patients with TNBC at initial diagnosis and the overall study population, with key SG-related AEs being hematologic toxicities and diarrhea [26].

Approximately one-third of patients in the ASCENT trial did not have TNBC at their initial breast cancer diagnosis. This finding is consistent with previous reports documenting changes in HER2 and Hr status over the course of disease, particularly at disease relapse or metastasis [13–15]. Loss of Hr expression following relapse is particularly common, occurring in approximately 25–45% of patients who have relapse of their primary tumor [13, 15]. The underlying reasons for changes in receptor status between primary and recurrent lesions may include intratumoral heterogeneity, changes in tumor biology, and selective pressure from previous therapies [14, 29, 30]. In patients who received trastuzumab as part of neoadjuvant therapy for HER2-positive breast cancer and did not achieve a pathogenic CR, approximately one-third of assessable residual tumors lost HER2 amplification [29]. Similarly, loss of PD-1/PD-L1 expression from primary to metastatic tumors is frequent, and resistance to immune checkpoint inhibitors is a concern [31, 32]. These studies indicate that residual or metastatic tumors should be reassessed for biomarker status, and novel treatment strategies like SG are needed in populations with altered biomarker status.

The subset of patients without TNBC at initial diagnosis in ASCENT represent a particularly heavily pre-treated population; these patients received a median of 5 prior anticancer regimens in any treatment setting for breast cancer, including endocrine therapy and everolimus, numerically higher than the four median prior regimens observed for patients with TNBC at initial diagnosis [26]. However, the clinical benefit with SG over TPC in patients without TNBC at initial diagnosis was similar to that observed for patients with TNBC at initial diagnosis and the overall ASCENT primary analysis population [26]. Although patients without TNBC at initial diagnosis who received prior CDK4/6 inhibitors and received SG had a numerically lower ORR (21%) compared with all patients without TNBC at initial diagnosis who received SG (31%) and the overall ASCENT primary analysis population (35%) [26], the numerically higher ORR in the SG versus TPC arms (21% vs. 5%) suggests that SG may have a clinical benefit in patients without TNBC who previously received CDK4/6 inhibitors.

The results of the current analysis are similar to those of the phase 1/2 IMMU-132-01 basket trial of SG for patients with breast cancer subtypes other than TNBC [33]. Like patients in ASCENT who did not have TNBC at initial diagnosis, the 54 patients in the earlier trial with Hr-positive, HER2-negative metastatic breast cancer were heavily pre-treated, and included CDK4/6 inhibition (59%). The ORRs and CBRs seen with SG in the phase 1/2 trial were 31% and 44%, respectively, with a median PFS of 5.5 months and median OS of 12 months, in line with the results observed in this analysis [33].

This subgroup analysis had several limitations. Primarily, the ASCENT trial was not designed to assess the efficacy of SG in patients without TNBC at initial diagnosis. Further, tumor phenotyping was not performed centrally on the initial breast cancer diagnostic tissue, or on the trial-qualifying tissue. As a result, information on specific changes in receptor status prior to enrollment in ASCENT are not available, limiting our interpretation of the efficacy and safety of SG for different subtypes of breast cancer. However, 27% versus 29% and 20% versus 17% of patients without TNBC at initial diagnosis in the SG versus TPC arms received prior CDK4/6 inhibitor and anti-HER2 therapy, respectively, suggesting a substantial proportion of patients in ASCENT may have had HER2-positive or Hr-positive disease prior to TNBC diagnosis. Additionally, the limited number of patients without TNBC at initial diagnosis enrolled in the ASCENT study, particularly those who also received prior CDK4/6 inhibitor therapy (SG, *n* = 19; TPC, *n* = 22), limits interpretability of these results.

In conclusion, this subanalysis from the ASCENT study showed that SG provides clinical benefit for patients with TNBC regardless of subtype at initial diagnosis, with a manageable safety profile. With the advent of new systemic treatment options for advanced TNBC, such as SG, patients with advanced disease should be reassessed for changes in breast cancer subtype to determine the optimal treatment. The results provide evidence for further evaluating SG as a treatment option for patients with subtypes other than TNBC, including those who previously received CDK4/6 inhibitors. However, additional studies are needed to further determine the efficacy and safety profile of SG in breast cancer subtypes other than TNBC. Ongoing studies include a phase 3 trial for Hr-positive, HER2-negative metastatic breast cancer (TROPiCS-02, NCT03901339) and multiple trials evaluating SG as a single-agent or in combination with other therapies for TNBC and HER2-negative breast cancer, including in the curative setting.

## Supplementary Information

Below is the link to the electronic supplementary material.

Supplementary file1 (PDF 194 kb)

## Data Availability

Immunomedics, Inc., a subsidiary of Gilead Sciences, Inc. will provide the study protocol and statistical analysis plan with publication of this manuscript as well as post results on clinicaltrials.gov, as required.
