# Supplementary material for: Analysis of patients without and with an initial triple-negative breast cancer diagnosis in the phase 3 randomized ASCENT study of sacituzumab govitecan in metastatic triple-negative breast cancer
Source: Breast Cancer Res Treat. 2022 May 11;195(2):127–39. doi: 10.1007/s10549-022-06602-7 (PMC9374646; doi:10.1007/s10549-022-06602-7)
Supplement: Supplementary file 1 — Supplementary file1 (PDF 194 kb) [file 10549_2022_6602_MOESM1_ESM.pdf]

SUPPLEMENTARY INFORMATION

Title: Analysis of patients without and with an initial triple-negative breast cancer diagnosis in the phase 3 randomized ASCENT study of sacituzumab govitecan in metastatic triple-negative breast cancer

Authors: Joyce O'Shaughnessy,[1] Adam Brufsky,[2] Hope S. Rugo,[3] Sara M. Tolaney,[4] Kevin Punie,[5] Sagar Sardesai,[6] Erika Hamilton,[7] Delphine Loirat,[8] Tiffany Traina,[9] Roberto Leon-Ferre,[10] Sara A. Hurvitz,[11] Kevin Kalinsky,[12]* Aditya Bardia,[13] Stephanie Henry,[14] Ingrid Mayer,[15] Yanni Zhu,[16] See Phan,[17] Javier Cortés[18]

Affiliations:

[1]Medical Oncology, Texas Oncology - Baylor Charles A. Sammons Cancer Center, Dallas, TX, USA; [2]Magee-Womens Hospital and the Hillman Cancer Center, University of Pittsburgh Medical Center, Pittsburgh, PA, USA; [3]Department of Medicine, University of California San Francisco Helen Diller Family Comprehensive Cancer Center, San Francisco, CA, USA; [4]Medical Oncology, Dana-Farber Cancer Institute, Boston, MA, USA; [5]Department of General Medical Oncology and Multidisciplinary Breast Centre, Leuven Cancer Institute, University Hospitals Leuven, Leuven, Belgium; [6]The Ohio State University Comprehensive Cancer Center, Columbus, OH, USA; [7]Sarah Cannon Research Institute/Tennessee Oncology, Nashville, TN, USA; [8]Medical Oncology Department and D3i, Institut Curie, Paris, France; [9]Memorial Sloan Kettering Cancer Center, New York, NY, USA; [10]Department of Oncology, Mayo Clinic, Rochester, MN, USA; [11]Medical Oncology, University of California, Los Angeles, Jonsson Comprehensive Cancer Center, Los Angeles, CA, USA; [12]Columbia University Irving Medical Center, New York, NY, USA; [13]Department of Hematology/Oncology, Massachusetts General Hospital Cancer Center, Harvard Medical School, Boston, USA; [14]Department of Oncology-Hematology, Radiotherapy, and Nuclear Medicine, CHU UCL Namur, Namur, Belgium; [15]Division of Hematology/Oncology, Breast Cancer Program, Vanderbilt-Ingram Cancer Center, Nashville, TN, USA; [16]Department of Biostatistics, Gilead Sciences Inc, Foster City, CA, USA; [17]Department of Clinical Development, Gilead Sciences Inc, Foster City, CA, USA; [18]International Breast Cancer Center, Quironsalud Group, Barcelona, Spain.

*Now affiliated with Winship Cancer Institute, Emory University, Atlanta, GA, USA.

Corresponding author:

Joyce O'Shaughnessy
Address: Medical Oncology, Texas Oncology-Baylor Charles A. Sammons Cancer Center
3410 Worth St., Suite 400, Dallas, TX 75246
Email: joyce.oshaughnessy@usoncology.com

Journal: *Breast Cancer Research and Treatment*

Online Resource 1. CONSORT diagram of patients without and with TNBC at initial diagnosis in the ASCENT study

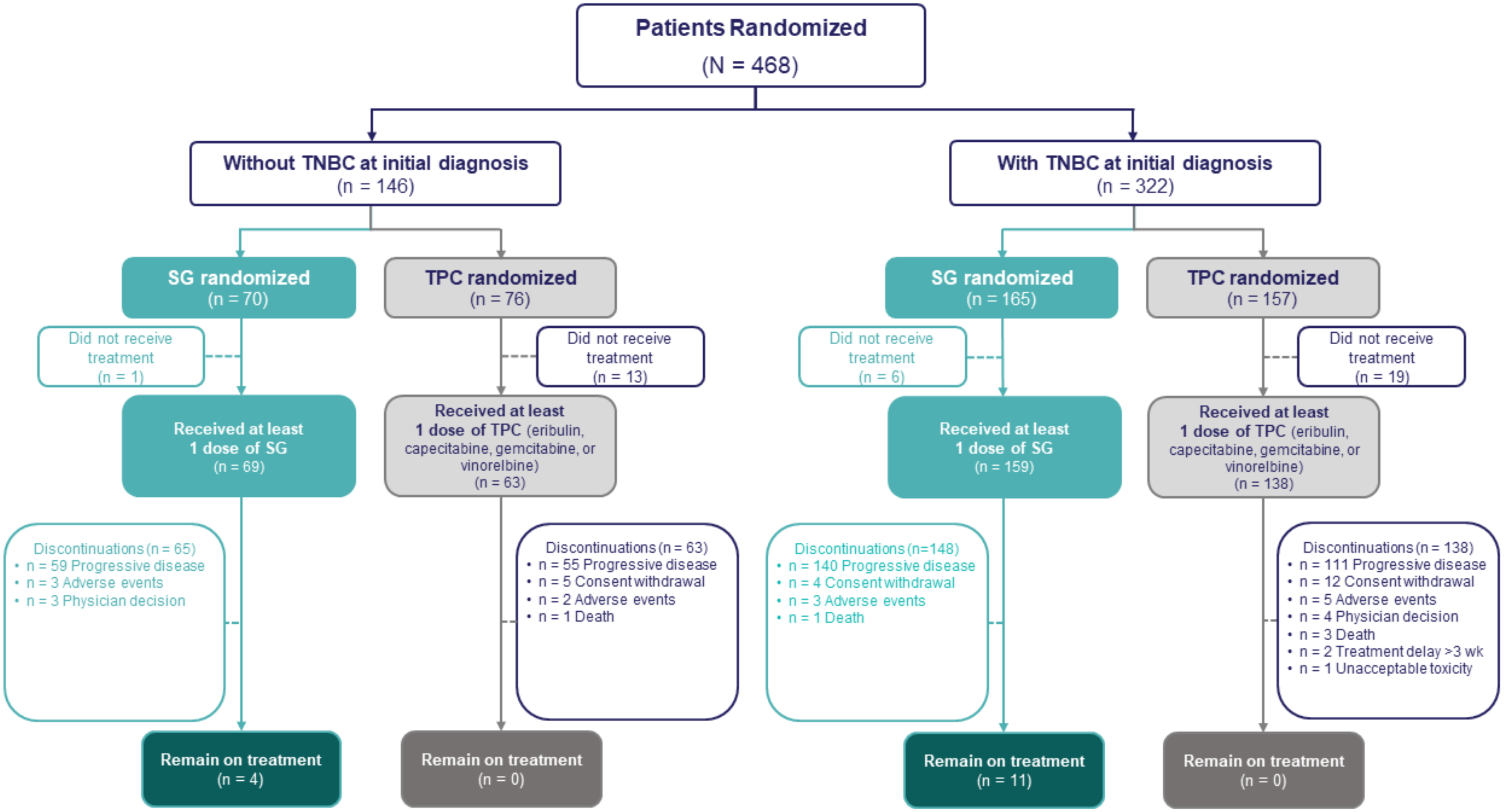

Patient disposition in the brain metastasis-negative population is shown.
SG, sacituzumab govitecan; TNBC, triple-negative breast cancer; TPC, treatment of physician's choice
